# Inflammation and Epstein-Barr Virus Infection Are Common Features of Myasthenia Gravis Thymus: Possible Roles in Pathogenesis

**DOI:** 10.4061/2011/213092

**Published:** 2011-09-26

**Authors:** Paola Cavalcante, Lorenzo Maggi, Lara Colleoni, Rosa Caldara, Teresio Motta, Carmelo Giardina, Carlo Antozzi, Sonia Berrih-Aknin, Pia Bernasconi, Renato Mantegazza

**Affiliations:** ^1^Department of Neurology IV, Neuromuscular Diseases and Neuroimmunology, Neurological Institute C. Besta Foundation, 20133 Milan, Italy; ^2^Anatomia Patologica, Azienda Ospedaliera Bolognini Seriate, 24068 Seriate, Italy; ^3^Unité Mixte de Recherche, CNRS UMR7215/INSERM U974/UPMC/AIM, Thérapie des Maladies du Muscle Strié, Centre Chirurgical Marie Lannelongue, 92350 Le Plessis Robinson, France

## Abstract

The thymus plays a major role in myasthenia gravis (MG). Our recent finding of a persistent Epstein-Barr (EBV) virus infection in some MG thymuses, combined with data showing that the thymus is in a proinflammatory state in most patients, supports a viral contribution to the pathogenesis of MG. Aim of this study was to gain further evidence for intrathymic chronic inflammation and EBV infection in MG patients. Transcriptional profiling by low density array and real-time PCR showed overexpression of genes involved in inflammatory and immune response in MG thymuses. Real-time PCR for EBV genome, latent (EBER1, EBNA1, LMP1) and lytic (BZLF1) transcripts, and immunohistochemistry for LMP1 and BZLF1 proteins confirmed an active intrathymic EBV infection, further supporting the hypothesis that EBV might contribute to onset or perpetuation of the autoimmune response in MG. Altogether, our results support a role of inflammation and EBV infection as pathogenic features of MG thymus.

## 1. Introduction

Myasthenia gravis (MG) is a well-characterized autoimmune disorder of the neuromuscular junction. In most cases (>80%), the disease is associated with the production of autoantibodies against the acetylcholine receptor (AChR), which impair neuromuscular transmission resulting in muscle weakness and disabling fatigability. Less frequently, MG is associated with the presence of antibodies against the muscle specific kinase (MuSK) receptor [1]. The remaining MG patients—referred as seronegative—are negative for anti-AChR and anti-MuSK antibodies, although a proportion of them (66%) has recently been found to have low-affinity anti-AChR antibodies [2].

A wealth of data supports the involvement of thymus in the pathogenesis of MG with AChR autoantibodies. Marked pathological alterations of thymus occur in over 80% of AChR-positive patients [1], comprising thymic hyperplasia observed in 50–60% of AChR-positive cases and variable proportion of seronegative cases [3–5], and thymoma present in 10–15% of cases. Thymus with hyperplasia contains B-cell infiltrates that can organize into ectopic germinal centers (GCs) forming B-cell follicles (follicular hyperplasia) or be distributed throughout thymic medulla (diffuse hyperplasia, also called thymitis) [3]. Ten to 20% of AChR-positive cases have an atrophic thymus very similar to that of age-matched controls with regard to the amount of adipose tissue and epithelial space and characterized by the presence of infiltrating B cells, in some cases forming GCs in the residual islands of medullary parenchyma [3, 4, 6], indicative of thymic hyperplasia and immune activation. 

The thymus of AChR-positive MG patients contains all the components required to initiate and sustain the autoimmune response: the autoantigen, expressed on muscle-like myoid cells [7] and thymic epithelial cells (TECs) [8], professional antigen-presenting cells [9], AChR-specific T cells [10], and plasma cells producing anti-AChR antibodies [11]. As sign of thymic involvement in MG pathogenesis, thymectomy results in stable remission in a high proportion of AChR-positive patients (see [12] and references included).

Both genetic and environmental factors are involved in the etiology of MG. Viral infections are the prime environmental factors suspected to play a role in the development of autoimmunity through mechanisms which include general activation of the host immune system and molecular mimicry [13]. In the former process, pathogens act as promoters of autosensitization mainly by initiating an innate immune response that in turn stimulates inflammation and activates the host immune system [13]. Striking evidence of chronic inflammation of thymus in most MG patients [14, 15] makes plausible the hypothesis that persistent viruses or other microbial agents may contribute to intrathymic etiologic mechanisms of the disease. Our recent findings provided indication of a viral contribution to onset or maintenance of the intrathymic autoimmune response in MG patients [6, 16]. In a study, we found evidence of a chronic poliovirus infection in the thymus of some (14.7%) MG patients, suggesting that persisting viruses, which stimulate innate immune responses and chronic inflammation, might be responsible for immunological alterations and autosensitization in the thymus [16]. In another study, we identified an abnormal accumulation of Epstein-Barr virus- (EBV-) infected B cells and plasma cells in MG thymuses but not in normal control thymuses [6]. We found viral DNA and both viral latency and lytic gene mRNAs and proteins in most of the examined MG thymuses, indicating EBV persistence and reactivation [6]. Since EBV has the unique ability to disrupt B-cell regulatory checkpoints and to interfere with the B-cell differentiation program [17, 18], our finding suggested that EBV infection may contribute to chronic B-cell activation and persistent autoimmune response in this organ in MG patients [6]. 

Herein, we searched for new evidence of inflammation and EBV infection in MG thymus. Our objectives were (a) to characterize MG thymus for the expression of genes involved in biological processes related to immune response, including genes encoding for proinflammatory molecules, regulators of immune response, and antiviral agents; (b) to gain further evidence of EBV infection in MG thymus by extending the search for EBV presence from the 17 MG thymuses examined in our previous study [6] to an additional 19 MG thymuses.

## 2. Material and Methods

### 2.1. MG Patients, Thymic Tissues, and Control Cell Lines

The study included pathological thymuses from MG patients who underwent thymectomy as therapeutic treatment and nonpathological thymuses obtained during heart surgery in babies and adult cardiopathic subjects. Written informed consent was obtained from all patients for thymectomy and use of thymus for research purposes. The study was approved by the Ethics Committee of the Carlo Besta Neurological Institute.

Thymic tissues from 10 AChR-positive MG patients (Patient Group 1), including 3 with follicular hyperplasia, 3 with thymitis, and 4 with thymic involution (see Table 1 for clinical features), were individually used for TaqMan Low-Density Array (LDA) Immune Panel analysis. Two nonpathological thymuses from babies aged 0.5 and 10 months were used as reference tissues in the LDA analysis. Real-time RT-PCR, performed to confirm LDA data, was carried out on MG thymuses from the 10 patients included in the LDA and from additional 17 patients (Patient Group 2, Table 1), including 6 with follicular hyperplasia, 6 with thymitis, and 5 with thymic involution, previously resulted positive for intrathymic EBV infection [6]; as control, the 2 nonpathological thymuses examined by LDAs and additional 5 EBV-negative nonpathological thymuses (mean age: 31.4 ± 17.3) [6] were analyzed. 

Real-time PCR for EBV DNA and RNA detection was carried out on thymuses from 19 MG patients (Patient Group 3, Table 1), including 15 AChR-positive MG patients (6 with follicular hyperplasia, 4 with thymitis, and 5 with thymic involution) and 4 seronegative MG patients (3 with follicular hyperplasia and 1 with thymitis). Thymuses from 2 adult healthy subjects, previously resulted EBV-negative [6], were analyzed to test the specificity of the PCR procedures described in what follows. 

For each thymus, some fragments were fixed in 10% formalin for histopathological classification; other fragments were snap-frozen and stored at −80°C. EBV-positive lymphoblastoid JY and EBV-negative human Jurkat T-cell lines were cultured at 37°C in 5% CO_2_ in RPMI 1640 (Euroclone, Pero, Italy) with 10% foetal bovine serum (Invitrogen, Carlsbad, CA), 2 mM sodium pyruvate (Invitrogen), 2 mM L-glutamine, and 100 U penicillin/streptomycin (all from Euroclone) and used as controls in molecular analyses and immunohistochemistry.

### 2.2. Transcriptional Profiling

#### 2.2.1. RNA Isolation and cDNA Synthesis

Total RNA was extracted by 20–50 mg of frozen thymic fragments using the TRIzol method (Invitrogen) and treated with DNase I (Ambion Applied Biosystems, Foster City, CA). Random-primed cDNA was prepared using Superscript II reverse transcriptase (Invitrogen) following the manufacturer's instructions. As a control of retrotranscription efficiency, *β*-actin gene was amplified in the same samples.

#### 2.2.2. TaqMan Low-Density Array (LDA)

cDNAs prepared from 10 MG thymuses (Patient Group 1) and 2 control thymuses were analysed by TaqMan Low-Density Immune Profiling Array, product number 4342510 (Applied Biosystems), a microfluidic card containing predesigned primer probe sets specific for 90 genes, implicated in the immune response (e.g., cytokines/chemokines and their receptors, transcription factors, stress response, cell surface receptors, and signal transduction), and for 6 housekeeping genes (e.g., *β*-actin and glyceraldehyde 3-phosphate dehydrogenase, GAPDH). To run the array, the cDNA was added to the PCR master mix (Applied Biosystems) and loaded into the eight sample-loading channels of LDA. Each channel with 48 wells contains primer probe sets for 12 different genes tested in quadruplicate. After a brief centrifugation, the arrays were run on an upgraded Applied Biosystems 7900HT Real-Time PCR System (performed at Cogentech, Consortium for Genomic Technologies c/o IFOM-IEO Campus, Milan, Italy). GAPDH was used to normalize the results. For each target gene, relative expression was calculated from the formula 2^−∆∆Ct^ using as calibrators normalized values obtained from control thymuses.

#### 2.2.3. Real-Time RT-PCR

cDNA samples prepared from thymic fragments of Patient Group 1 and 2, and 7 control thymuses, were subjected to real-time PCR for IL-6, IL-10, IFN-1*β*, IFN-*γ*, MxA, and HLA-DR*α* genes. Predesigned functionally tested TaqMan gene expression assays (Applied Biosystems) were used: assay ID Hs00174131_m1 for IL-6; assay ID Hs00174086_m1 for IL-10; assay ID Hs01077958_s1 for IFN-*β*; assay ID Hs00174143_m1 for IFN-*γ*; assay ID Hs00182073_m1 for MxA; assay ID Hs00740413_g1 for HLA-DR*α*. Each cDNA was amplified in triplicate using 7500 Fast Real-time PCR system (Applied Biosystems) in a PCR volume of 20 *μ*L containing 10 *μ*L of TaqMan Fast Universal PCR Master Mix and 1 *μ*L of TaqMan gene expression assays (all from Applied Biosystems). GAPDH mRNA was analyzed as endogenous control by using TaqMan Predeveloped Assay Reagents Human GAPDH (Applied Biosystems). The omission of cDNA was taken as no template control. Data analysis followed the same method (2^−ΔΔCt^ method) as that in LDA.

#### 2.2.4. Statistical Analysis

One-way ANOVA with Bonferroni multiple comparison post hoc test was performed to assess the significance of differences in transcriptional profiling LDA data. In real-time RT-PCR analysis, Mann-Whitney *U* test was used to compare IL-6, IL-10, IFN-*β*, IFN-*γ*, MxA, and HLA-DR*α* transcript levels in control and MG thymus. *P* values < 0.05 were considered significant. GraphPad PRISM version 4.0 (GraphPad Software, San Diego, CA) was used for data elaboration and statistical analysis.

### 2.3. Tissue Processing for EBV Detection

#### 2.3.1. DNA and RNA Isolation

For DNA and RNA isolation aimed to detect EBV genome and transcripts, snap-frozen thymic specimens from the donors belonging to the Patient Group 3 and from 2 control thymuses were used. For each OCT-included snap-frozen thymus, a total of 18 serial sections were obtained using a cryostat (Leica Microsystems, Nußloch, Germany) for alternate DNA, RNA isolation, or immunohistochemistry. 30-*μ*m sections 1, 4, 7, 10, 13, and 16 were collected for DNA extraction, 30-*μ*m sections 3, 6, 9, 12, 15, and 18 for RNA extraction, and 6-*μ*m sections 2, 5, 8, 11, 14, and 17 for immunohistochemistry.

For DNA isolation, thymic sections were resuspended in 300 *μ*g/mL proteinase K in digestion buffer, homogenized with TissueLyser LT (Qiagen, Valencia, CA), and incubated at 50°C overnight; DNA was extracted following the standard phenol-chloroform protocol. RNA was isolated from thymic sections by using the TRIzol method (Invitrogen), after homogenization with TissueLyser LT (Qiagen). RNA integrity was checked on ethidium bromide containing 1% agarose gel in Tris-borate/EDTA buffer. All RNA samples were treated with DNase I (Ambion Applied Biosystems). Concentration of DNA and RNA was estimated by Nanodrop 2000 c Spectrophotometer (Thermo Fisher Scientific, Wilmington, DE).

#### 2.3.2. Real-Time PCR for EBV DNA

Real-time PCR specific for the *Bam*HI-W repeated multiple splices [19] was performed on each DNA sample. Genomic DNA (0.8 *μ*g) was amplified in a final volume of 25 *μ*L containing 12.5 *μ*L of TaqMan Universal PCR Master Mix (Applied Biosystems), 900 nM each primer, and 175 nM probe. Primers and probe used were as in [19]. Following two steps at 50°C for 2 min and 95°C for 10 min, 50 cycles of 1 sec at 95°C and 1 min at 60°C were carried out by a 7500 Fast Real-time PCR System (Applied Biosystems). Real-time PCR reactions were performed in duplicate, including a no template control consisting in the omission of DNA. A threshold cycle (Ct) value was calculated by determining the point at which the fluorescence exceeded a threshold limit (10 times the standard deviation of the baseline). Samples were defined positive for Ct values lower than 38 cycles. DNA integrity and amplification efficiency was checked by amplifying a fragment of the *β*-globin gene from each DNA preparation. 

To test sensitivity and efficiency of the real-time PCR assay, dilution series (0.5 to 5 × 10^3^ copies of EBV genome) of the DNA isolated from the EBV-positive JY cells [20] were analyzed in triplicate. The standard curve was obtained automatically by using the 7500 Fast System software. As negative control, DNA derived from Jurkat cells was amplified.

#### 2.3.3. Real-Time RT-PCR for EBV Latency Transcripts

Real-time RT-PCR for the detection of EBV-encoded small RNA (EBER) 1, EBV nuclear antigen (EBNA) 1, and latent membrane protein (LMP) 1 transcripts was performed on DNase-treated RNA (0.5 *μ*g) from thymic sections. TaqMan PCR primers and probes for EBER1, EBNA1, and LMP1 were as in [19, 21]. EBNA1 and LMP1 primers and probes were incorporated into TaqMan Gene Expression Assays by Applied Biosystems. RNA was amplified in a final volume of 20 *μ*L containing 5 *μ*L of 4x TaqMan Fast Virus 1-Step Master Mix and 1 *μ*L of TaqMan Gene Expression Assay for EBNA1, LMP1, and GAPDH or 1.25 *μ*M each primer and 0.18 *μ*M probe for EBER1 (all from Applied Biosystems). TaqMan Fast Virus 1-Step Master Mix (Applied Biosystems) is designed for high-sensitivity virus detection and performs reverse transcription and PCR all in one reaction. 

Real-time RT-PCR reactions were incubated on 7500 Fast Real-Time PCR System (Applied Biosystems) at 50°C for 5 min and 95°C for 15, followed by 50 cycles at 95°C for 15 sec and 60°C for 1 min. Real-time RT-PCR reactions were performed in duplicate, including a no template control consisting in the omission of RNA. Detection of GAPDH transcript (Applied Biosystems) served as control for the presence of template RNA and efficiency of real-time RT-PCR. A threshold cycle (Ct) value was calculated as described above for EBV DNA detection. Samples were defined positive for Ct value lower than 38 cycles.

To test sensitivity and efficiency of the real-time RT-PCR assays, dilution series (from 0.1 to 10^5^ cells per reaction) of the RNA obtained from the EBV-positive cell line JY were amplified in presence and absence of 1 *μ*g of RNA from the EBV-negative Jurkat T-cell line. The standard curve was obtained automatically by using the 7500 Fast System software. As negative control, RNA derived from Jurkat T cells was amplified. Each point of standard curve was run in triplicate. PCR product identity was checked by sequencing on an ABI 3100 Genetic Analyzer (Applied Biosystems).

#### 2.3.4. Real-Time RT-PCR for BZLF1 Lytic EBV Transcript

For the detection of EBV lytic transcript BZLF1, DNase-treated RNA obtained from thymic sections was retrotranscribed into random-primed cDNA by using SuperScript Vilo cDNA Synthesis kit (Invitrogen). cDNA corresponding to 500 ng of RNA was amplified with the BZLF1-out forward and reverse primers previously reported [22]. Amplification was performed in a final volume of 50 *μ*L consisting of 1x PCR buffer (Finnzyme, Espoo, Finland), 0.2 mM dNTPs (Applied Biosystems), 0.4 *μ*M of each primer, and 1 U of DNAZyme (Finnzyme). After a predenaturation step at 95°C for 5 min, 40 cycles were repeated at 95°C for 1 min and 59°C for 1 min followed by an extension step of 7 min at 72°C. For each sample, 5 *μ*L of PCR product were subjected to real-time PCR in a volume of 20 *μ*L containing 10 *μ*L of *Power *SYBR Green PCR Master Mix (Applied Biosystems) and 0.8 *μ*M each of BZLF1-inn primers [22] and incubated on 7500 Real-time PCR System (Applied Biosystems). As a control of retrotranscription efficiency, *β*-actin gene was amplified in the same samples. cDNA from JY and Jurkat cell lines was amplified as positive and negative control, respectively, and PCR product identity was checked by sequencing on an ABI 3100 Genetic Analyzer (Applied Biosystems).

#### 2.3.5. Immunohistochemistry

Immunohistochemistry was performed on 6-*μ*m sections from snap frozen thymic tissues belonging to Patient Group 3 (*n* = 19). All the 19 thymuses were immunostained with antibodies specific for human CD20 (1 : 300; clone L26, Dako, Glostrup, Denmark) and CD138 (1 : 50; clone MI15, Dako). Sections from 8 MG thymuses (4 follicular hyperplasia, 2 thymitis, and 2 involuted thymuses) were immunostained with antibodies for latent EBV protein LMP-1 (ready-to-use, clone CS 1-4, isotype IgG1, Dako) and lytic EBV protein BZLF1 (1 : 10; isotype IgG2, Lifespan Biosciences Inc., Seattle, WA). Sections were fixed with 4% paraformaldehyde and incubated for 10 min in 1.5% hydrogen peroxide in methanol, to eliminate endogenous peroxidase activity. For BZLF1 immunostaining, sections were treated with 0.1%Triton X 100 for 10 min. To block nonspecific binding, sections were incubated for 1 hour in 5% BSA. Incubations with primary antibodies were performed overnight at 4°C. Sections were then incubated with DakoCytomation EnVision + System Labelled Polymer-HRP Anti-Mouse (Dako) for 1 hour. Peroxidase reaction was visualized with 3,3′ diaminobenzidine (DAB) plus substrate buffer (Dako). All sections were counterstained with hematoxylin, visualized by optical microscopy (Nikon, Germany), and examined using Image Proplus (Media Cybernetics, Silver Spring, MD). Immunohistochemistry specificity was controlled by omitting the primary antibodies or replacing them with isotype-specific nonimmune IgG (Dako).

## 3. Results

### 3.1. Transcriptional Profiling of MG Thymus

To characterize the thymic transcriptome of MG patients for the expression of genes implicated in inflammation and immune response, we preliminarily used LDA approach on a small series of MG patients and found that gene expression profile of MG thymuses were distinct from that of control thymuses, delineating a thymic condition characterized by chronic inflammation and active immune response, as previously reported [14, 15]. Starting from our LDA results and from the published data [14, 15], we selected six genes (IL-6, IFN-*γ*, HLA-DR*α*, IL-10, IFN-*β*, and MxA), expected to be dysregulated during inflammation and immune response, and analysed their expression by real-time RT-PCR on a higher number of MG and control thymuses, including EBV-positive MG thymuses and EBV-negative nonpathological thymuses [6]. The results confirmed inflammatory state and immune response activation in MG thymus. LDA and real-time RT-PCR results are described in detail in what follows. 

#### 3.1.1. LDA Data Reflect Inflammatory State and Immune Activation in MG Thymus

The ninety genes included in the LDA assays were successfully amplified in MG and control thymuses. The analysis of variance identified 21 genes whose expression was significantly different among the MG and control sample groups (*P* < 0.05). The other genes were expressed at similar level in all groups except some of them (e.g., Bcl-2-like protein 1, a potent inhibitor of cell death; angiotensin II type-2 receptor, a protein belonging to the G-protein coupled receptor 1 family involved in programmed cell death; CD38, a transmembrane glycoprotein involved in cell adhesion, signal transduction, and calcium signalling) whose expression levels were lower in MG thymuses compared to controls, although the differences were not statistically significant. In Table 2, we reported LDA data for genes that were significantly upregulated in at least one of the three MG thymus subgroups versus normal thymuses. MG thymuses with thymitis showed the highest number of upregulated genes, prevalently cytokines and chemokines (Table 2). Among cytokines, IL-6—a well-known highly inflammatory cytokine implicated in chronic inflammatory and autoimmune diseases [23]—was the most overexpressed gene in each MG thymus subgroup compared to normal thymus, followed by IL-1*β*, a proinflammatory cytokine mainly produced by myeloid cells and also involved in various inflammatory and autoimmune diseases [24] (Table 2). Transcriptional levels of other molecules able to modulate inflammatory process, including colony-stimulating factor (CSF) 1, IL-7, IL-10, IL-12p35, TNF-*α*, and IFN-*γ*, were also higher in MG thymic conditions with respect to control thymuses (Table 2). A significant overexpression of IL-10—a Th2-produced cytokine having inhibitory properties on Th1 function and promoting humoral immune response [25, 26]—was identified whatever MG thymic subgroup was considered (Table 2).

Among chemokines, the chemokine receptor CXCR3, involved in recruitment and maintenance of activated T cells in the inflammatory site [27], was significantly upregulated in follicular hyperplasia and thymitis (Table 2), according to previous data [28]. Transcriptional levels of RANTES (CCL5), IL-8, monocyte chemoattractant protein- (MCP-) 1, macrophages inflammatory protein- (MIP-) 1*α*—monocyte chemotactic factors that are highly produced during microbial infection [29]—were increased in each thymic MG subgroup (Table 2).

We found that mRNA level of vascular endothelial growth factor A (VEGF-A)—a growth factor mediating vascular permeability and vasculogenesis [30]—was significantly upregulated in thymitis compared with normal thymuses (Table 2).

Moreover, LDA identified upregulation in MG thymus of some genes involved in immune response and antigen presentation. As expected, we observed increased expression of CD19—marker of B cells—in each MG thymus subgroup (Table 2), reflecting B cell infiltration characteristic of MG thymus [3, 6]. Transcriptional level of CD152 (CTLA-4) was higher in MG than normal thymuses, especially in thymitis. CD152 is a surface molecule mostly considered as a negative regulator of T-cell activation [31]; recently, it has been demonstrated that CD152 signalling may play a role in antimicrobial infection by endowing effector T cells with the capacity to migrate to sites of inflammation and lymph nodes [32].

In line with previous data [15], we found that MG thymuses had high expression levels of HLA-DR*α* (Table 2) and complement component C3 mRNA levels. 

Among the 21 upregulated genes in MG thymus, there were also two genes, the SMAD family member 7 (SMAD7)—a nuclear regulator of transforming growth factor-*β* whose expression is altered in inflammatory diseases [33]—and the endothelial converting enzyme 1 (ECE)—a metalloprotease involved in proteolytic processing of endothelial precursors [34].

Differences between hyperplasia, thymitis, and involuted thymus were significant for CSF1, RANTES, VEGF-A, CXCR3, CD19, CD86, HLA-DR*α*, and MADH7 mRNAs, which were significantly higher in thymitis than involuted thymus; IL-1*β* and MCP-1 mRNAs, which were significantly higher in thymitis than hyperplasia; IL-7, IL-12p35, TNF-*α*, IFN-*γ*, IL-8, and CD*34* mRNAs, which were significantly higher in thymitis than hyperplasia and involuted thymus.

#### 3.1.2. Real-Time RT-PCR Shows Upregulation of Genes Involved in Inflammation and Antiviral Response in MG Thymus

Data derived from LDA were validated on a higher number of patients (Patient Group 2) and controls by conventional real-time PCR. We selected IL-6, IFN-*γ*, and HLA-DR*α* genes, known to be upregulated during inflammatory response against microbial infection and resulted upregulated in MG thymus by LDA (Patient Group 1, Table 1). We also investigated the expression of IL-10, for its role in modulating B cell function and humoral responses [26], type I IFN-*β*, which plays a pivotal role in the host immune response against viral infections [25], and MxA, an important mediator of type I IFNs [35].

**Figure 1:**
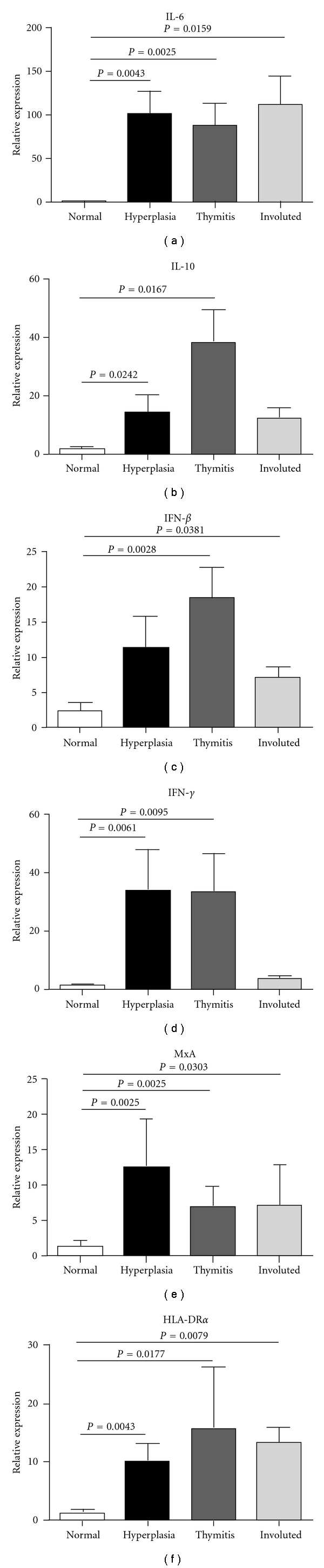
Relative expression of IL-6 (a), IL-10 (b), IFN-*β* (c), IFN-*γ* (d), MxA (e), and HLA-DR*α* (f) in the thymus of MG patients. The transcripts were analysed by real-time PCR analysis starting from total RNA extracted from the thymus of MG patients with hyperplasia (*n* = 9), thymitis (*n* = 9), and thymic involution (*n* = 9), and 7 healthy subjects. Relative expression of the 6 genes was normalized to GAPDH and calculated as 2^−ΔΔCt^; normalized values for nonpathological thymuses were used as calibrator. Values shown are means ± SEM of duplicate determinations. *P* values were obtained by the Mann-Whitney *U* test.

We found a significant upregulation of IL-6 in each MG thymus subgroup compared with normal thymuses (Figure 1(a)), confirming LDA data. An increased expression of IL-10 was also observed in all MG thymuses; in particular, IL-10 expression was significantly increased in hyperplasia and thymitis cases (Figure 1 (b)). Transcriptional level of IFN-*β* was significantly higher in thymitis and involuted thymuses (Figure 1(c)), whereas IFN-*γ* transcript was significantly up-regulated in hyperplasia and thymitis cases (Figure 1(d)). A significant increase in MxA expression was detected in each MG thymic subgroup, supporting the hypothesis of an ongoing antiviral response in MG thymus (Figure 1(e)). HLA-DR*α* also showed a significant upregulation in each MG thymic pathology, again supporting the inflammatory state of MG thymus (Figure 1(f)).

### 3.2. Characterization of MG Thymus for the Presence of EBV DNA, RNA, and Proteins

To confirm previous evidence of EBV infection in MG thymus [6], we investigated thymic tissues from 19 MG patients (Patient Group 3, Table 1) and two adult healthy donors for the presence of EBV DNA (*Bam*HI-W repeat region), RNA (EBER1, EBNA1, LMP1, and BZLF1), and proteins (LMP1 and BZLF1). 

#### 3.2.1. Presence of Infiltrating B Cells and Plasma Cells in MG and Control Thymuses

**Figure 2:**
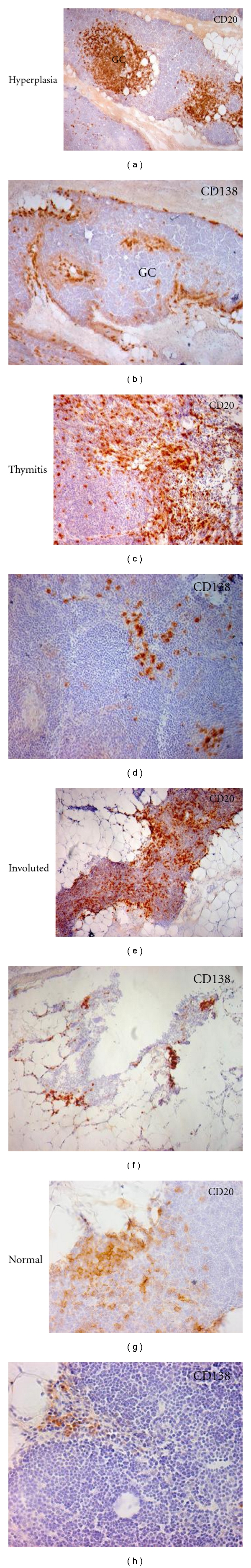
Presence of B cell lymphoid infiltrates and plasma cells in MG and control thymuses analysed for EBV detection. (a, b) Thymus with follicular hyperplasia (MG1). CD20+ B cells aggregate to form a germinal center (GC) in the thymic medulla (a). Many CD138+ plasma cells are present at the periphery of a GC (b). (c, d) Thymus with thymitis (MG11). Many CD20+ B cells (c) and CD138+ plasma cells (d) are sparse throughout the medullary infiltrates. (e, f) Involuted thymus (MG17). The residual thymic parenchyma contains lymphoid infiltrates with numerous CD20+ B cells (e) and CD138+ plasma cells (f). (g, h) Normal thymus from an adult healthy subject. Some CD20+ cells (g) and rare CD138+ plasma cells (h) are present in the thymic parenchyma. Magnifications: ×10 (a, b, e, f); ×20 (c, d); ×40 (g, h).

MG thymuses were initially examined for the presence of lymphoid B cell infiltrates and plasma cells, in order to verify that the thymic fragments under investigation contained B cells/plasma cells potentially positive to EBV. Presence of lymphoid B cell infiltrates (diffuse or organized in GCs) and plasma cells was found in all MG thymus specimens examined (Figures 2(a) to 2(f)); thus, alternate sections collected from these specimens were used for further analysis of EBV DNA and RNA presence.

#### 3.2.2. Detection of EBV DNA

**Figure 3:**
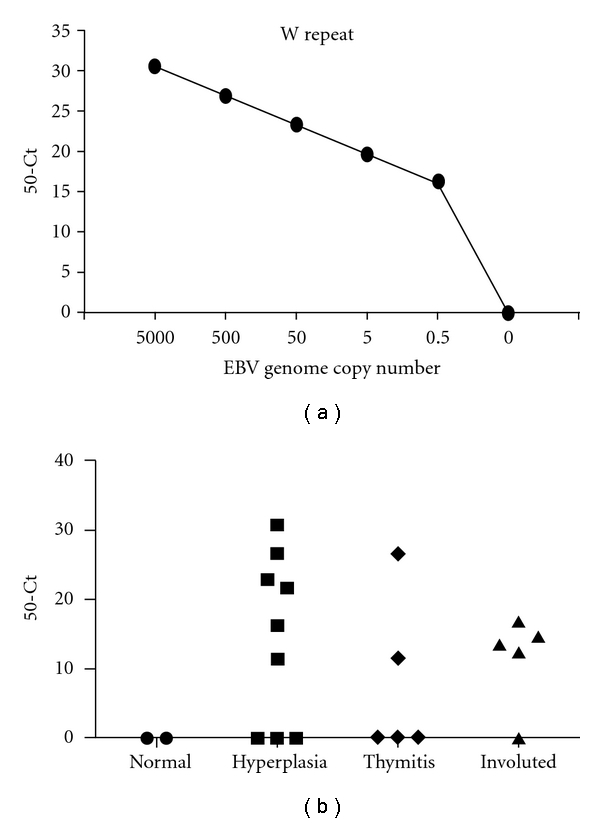
Real-time PCR for EBV genome detection (*Bam*HI-W repeats). (a) Dilution series of DNA (ranging from 0.5 to 10^5^ copies of EBV genome per reaction), extracted from the JY lymphoblastoid cell lines, were analysed as described in Section 2. Real-time PCR resulted in high and constant amplification efficiency for >0.5 copies of EBV genome per reaction. (b) Genomic EBV W repeats were detectable in 6/9 hyperplastic thymuses, 2/5 thymitis, and 4/5 involuted thymuses, but not in two nonpathological control thymuses. Real-time PCR was performed for 50 cycles, and results are expressed as 50-Ct.

We used real-time PCR to detect EBV genome (*Bam*HI-W repeat region) in MG thymuses. We first validated our real-time PCR assay by demonstrating that it was able to detect EBV DNA with high sensitivity and specificity. By amplifying dilution series of DNA from the EBV-positive JY cells [20], we obtained a standard curve showing linearity in the range from 0.5 to 5 × 10^3^ copies per reaction, with 0.99 regression coefficient (*R^2^*) and −3.56 slope, corresponding to 90.98% efficiency. We argued that the molecular system resulted in high and constant amplification efficiency and that >0.5 copy of genome for reaction could be quantified with an acceptable level of accuracy (Figure 3(a)). *β*-globin control gene was detected at similar level in all MG and control samples (data not shown). EBV genome was detected in 12/19 (63.2%) MG thymuses (6/9 hyperplasia, 2/5 thymitis, and 4/5 involuted thymuses) but not in control thymuses (Figure 3(b) and Table 3) and EBV-negative Jurkat T-cell line.

#### 3.2.3. Detection of Latent EBV Transcripts

**Figure 4:**
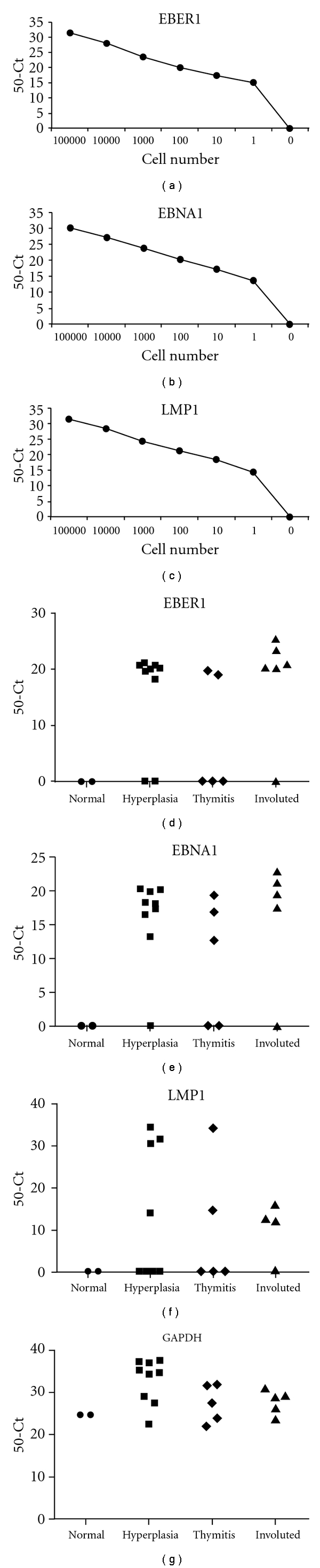
Real-time RT-PCR for the detection of latent EBER1, EBNA1, and LMP1 transcripts. (a, b, c) Analysis of sensitivity of the assay for EBER1 (a), EBNA1 (b), and LMP1 (c) showed that the three EBV latent transcripts could be detected in RNA extracted from a single JY EBV-infected cell. (d, e, f, g) EBER1 (d), EBNA1 (e), and LMP1 (f) were detected in most of the examined MG thymuses but not in normal control thymuses. All MG and control thymuses analysed showed high signals for endogenous control GAPDH amplification (g). Real-time RT-PCR was performed for 50 cycles, and results are expressed as 50-Ct.

We used real-time RT-PCR to analyse latent EBER1, EBNA1, and LMP1 transcripts in MG and control thymuses. We first validated our real-time RT-PCR assays by performing a number of control experiments. By using RNA from the EBV-positive JY cells, we demonstrated that our assays were able to detect the target transcripts in RNA from one EBV-positive JY cell for reaction (Figures 4(a) to 4(c)) but not in the EBV-negative Jurkat T-cell line. Standard curves for the detection of the three targets showed *R* ^2^ always higher than 0.99 and slope ranged from −3.22 and −3.47, corresponding to efficiencies higher than 94%. Real-time RT-PCR were also able to detect the target RNA from one EBV-positive JY cell in the presence of 1 *μ*g of RNA from the EBV-negative Jurkat T-cell line (~100,000 cells), indicating that our molecular systems could detect 1 positive cell/~100,000 negative cells. 

EBER1 (Figure 4(d)), EBNA1 (Figure 4(e)), and LMP1 (Figure 4(f)) transcripts were detected in 14/19, 15/19, and 9/19 MG thymuses, respectively, but not in control thymuses (Figures 4(d) to 4(f) and Table 3). Housekeeping gene GAPDH was detected in all the specimens analysed (Figure 4(g)).

#### 3.2.4. Detection of Lytic BZLF1 EBV Transcript

**Figure 5:**
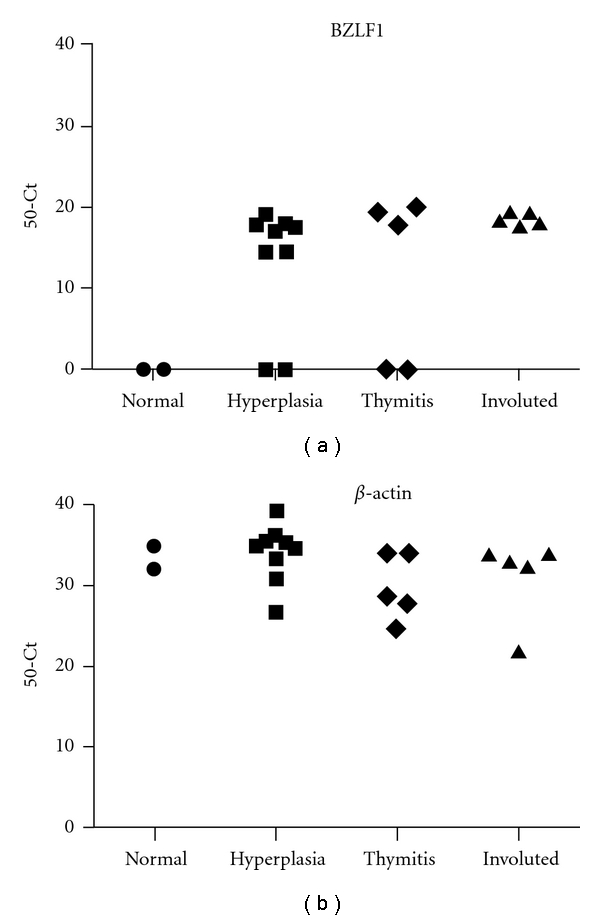
Real-time RT-PCR for the detection of lytic BZLF1 transcript. (a) Real-time RT-PCR for BZLF1 could detect the lytic transcript in 7/9 hyperplastic thymuses, 3/5 thymitis, and 5/5 involuted thymuses, but not in normal control thymuses. (b) All MG and control thymuses analysed showed high signals for endogenous control *β*-actin amplification.

BZLF1 transcript was detected in 16/19 MG thymuses but not in normal control thymuses (Figure 5(a)). In all MG and control cDNA specimens, the housekeeping gene *β*-actin was efficiently amplified (Figure 5(b)).

#### 3.2.5. Detection of Latent LMP1 and Lytic BZLF1 EBV Proteins

**Figure 6:**
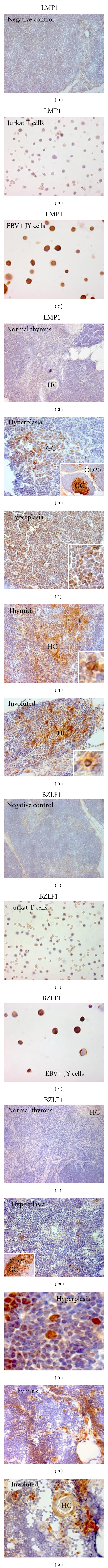
Immunohistochemistry for latent LMP1 (a)–(h) and lytic BZLF1 (i)–(p) EBV proteins. (a) to (h) LMP1 immunostaining. No signal of immunoreactivity was observed in negative control performed by incubating sections with isotype-specific nonimmune IgG (Dako) (a). LMP1 was not detected in EBV-negative Jurkat T-cell line (b) but was readily detectable in EBV-positive JY cells (c). Normal thymuses showed no immunoreactivity for LMP1 antibody (d). In hyperplastic thymuses (MG1 is shown), LMP1+ cells were detected in areas containing CD20+ B cells organized in germinal centers (GCs) ((e) and inset in (e)) or were diffused throughout the highly infiltrated medullary region (f). Numerous LMP1+ cells were identified in thymitis cases (MG13 is shown), that were diffused in thymic medulla and frequently located around Hassall's corpuscles (HCs), where often concentrate B cells (g). In involuted thymuses (MG16 is shown), numerous LMP1+ cells were scattered in the residual thymic parenchyma and frequently located in thymic infiltrated areas around HCs (h). Inset in (e) shows CD20 immunostaining of the same area of the main panel in a serial section. Insets in (f), (g), and (h) show areas of the main panels at higher power of magnification to reveal membrane localization of LMP1. (i) to (p) BZLF1 immunostaining. No signal of immunoreactivity was observed in negative control performed by incubating sections with isotype-specific nonimmune IgG (Dako) (i). BZLF1 was not detected in EBV-negative Jurkat T-cell line (j) but was readily detectable in EBV-positive JY cells (k). Normal thymuses showed no immunoreactivity for BZLF1 antibody (l). In hyperplasia (MG1 is shown), BZLF1+ cells were often detected at the edge of GCs ((m) and inset in (m)) or were scattered in thymic medulla (n). Inset in (m) shows CD20 immunostaining of the same area of the main panel in a serial section. In thymitis (MG11 is shown) (o) and involuted thymuses (MG17 is shown) (p), BZLF1+ cells were present in thymic medulla, in some cases located within medullary infiltrates in proximity to HCs. Magnifications: ×20 (a, d, i, l); ×40 (b, c, e, f, g, h, j, k, m, o, p); ×80 (n).

To confirm results of molecular analysis at protein level, we performed immunohistochemistry assays to detect LMP1 and BZLF1 proteins, markers of EBV latency and reactivation, respectively, on 8 MG and 2 control thymuses. Cells expressing LMP1 were detected in 7/8 MG thymuses analysed but not in normal thymuses (Figure 6 and Table 4). Immunoreactivity for LMP1 was mainly detected in GC and perifollicular areas in hyperplasia (Figures 6(e) and 6(f)) and within medullary infiltrates in thymitis and involuted thymuses (Figures 6(g) and 6(h)). We found cells expressing the early lytic phase EBV protein BZLF1 in thymic medulla of most MG thymuses examined (7/8) but not in normal thymuses (Figures 6(l) to 6(p) and Table 4), thus suggesting productive, not only latent, EBV infection in the thymus of MG patients.

## 4. Discussion

This study confirms and extends previous evidence of inflammation and viral infection in the thymus of MG patients.

### 4.1. Inflammation and Active Immune Response Characterize the Thymus of MG Patients

Previous analyses of the genes characterizing the hyperplastic thymus using microarray and real-time PCR approaches showed that transcripts of a large number of genes associated with inflammation and immune response were significantly upregulated in hyperplastic MG thymuses compared to controls [14, 15]. The upregulated genes included IFN-regulated genes, MHC Class II molecules, Ig family, and B cell-related genes, whose increase reflected an inflammatory state and a generalized B cell infiltration in hyperplastic MG thymus [14, 15]. 

In the present study, we used LDA approach to characterize the thymic transcriptome in 10 MG patients (Patient Group 1) whose thymuses had histopathological features of hyperplasia, thymitis, and thymic involution, and in non-MG subjects having normal thymuses.

LDA, a TaqMan quantitative PCR based on microfluidic systems, represents a valuable approach for sensitive and quantitative gene expression profiling that enables high throughput screening in functional genomics by simultaneously analysing mRNA expression of multiple genes in human tissues [36]. LDA technology allowed us to analyse mRNA of 90 genes belonging to different biological categories, including genes involved in inflammatory and immune responses. All the target transcripts were detected in MG and control thymuses. However, 21 genes were upregulated in MG thymuses compared to controls (Table 2): (a) proinflammatory cytokines, able to activate immune cells and having antiviral properties (i.e., IL-6, IL-1*β*, CSF, IL-7, IL12p35, TNF-*α*, and IFN-*γ*); (b) cytokines, chemokines, and molecules involved in migration, homing, and survival of lymphocytes in biological site of inflammation or infection (i.e., IL-6, RANTES, IL-8, MCP-1, MIP-1*α*, CXCR3, and CD152); (c) B-cell-related genes and genes related or potentially related to antigen presentation and humoral response (i.e., IL-10, HLA-DR*α*, CD19, and complement component C3).

The expression of proinflammatory cytokines reached the highest values in the thymitis cases (Table 2). Most of these cytokines are known to work in synergy and to promote inflammation and immune response during host defence, especially against viral infections [25]. Some of them are potent inflammatory molecules mainly involved in acute inflammation (i.e., IL-6, IL-1*β*, TNF-*α*, and CSF); others are mainly involved in establishing chronic inflammation and promoting humoral and cellular immune response (i.e., IL-7, IL-10, IL-12, and IFN-*γ*) [25]. Upregulation of IL-6 and the chemokine RANTES in MG compared to normal thymus was in line with previous studies showing that these genes were abnormally overexpressed in MG TECs either at basal condition [37] or (IL-6) when stimulated by lipopolysaccharide (LPS) [38], a major activator of Toll-like receptor (TLR) 4 known to be upregulated in MG thymus [39]. IL-6 is a well-known proinflammatory agent with pathological regulatory function on growth and differentiation of T- and B-cells [40]; RANTES has been observed to regulate the transepithelial migration of T cells [41]. Thus, overexpression of IL-6 and RANTES could support the migration of peripheral lymphocytes to thymus and their survival there, contributing to the pathological remodeling of the gland typical of MG [37]. Overexpression of IL-10 (Table 2) was also of pathogenic relevance, as IL-10 is a B-cell-related cytokine which modulates inflammatory processes and determines the antibody response by influencing B cell activation, proliferation, and differentiation [26]. IL-10 was found to be upregulated in serum of MG patients after immunoadsorption, indicating that this cytokine might be linked with the IgG synthesis or resynthesis process in MG [42]. Moreover, it has been shown that EBV infection through EBER signalling is able to induce the expression of IL-10 in B lymphocytes, where this cytokine could act as autocrine growth factor [43]. Thus, the observed IL-10 upregulation may be also explained by the previously observed intrathymic EBV infection in MG thymus [6] and implicated in B cell abnormalities which characterize this organ in MG patients. 

IFN-*γ* is a type II interferon which exhibits strong antiviral properties and ability to enhance MHC Class I and II expression on nucleated cells [25]. In our LDA data, both IFN-*γ* and HLA-DR*α* were upregulated in MG thymuses (Table 2), supporting an antiviral reaction and a local proinflammatory environment. Increased expression of MHC Class II molecules was previously observed by Le Panse and colleagues [15], whose results indicated that overexpression of these molecules in MG hyperplastic thymus was not directly related to the increased B cell number but could be due to the proinflammatory state of MG thymus.

Our transcriptome analysis showed also overexpression of IL-8, MCP-1, and MIP-1*α* (Table 2); these are inflammatory chemokines linked to innate immune responses and acting as leukocyte chemotactic factors [29]; their expression was higher in MG thymuses than in controls (Table 2), suggesting that they could be implicated in abnormal recruitment of T and B cells. Chemokine receptor CXCR3, known to drive migration and homing of activated T cells in inflammatory site [27], was particularly upregulated in hyperplasia and thymitis (Table 2) confirming previous observations of increased expression of CXCR3 and its ligand IFN-*γ*-inducible protein 10 in the thymus of MG patients [28].

The idea that thymic microenvironment in MG patients is favourable to abnormal migration of T cells is further supported by the observation that the expression of CD152 (CTLA-4) and its CD86 ligand was increased in MG thymuses compared to controls (Table 2). The CD152 molecule is generally considered as a negative regulator of T-cell activation [31]; however, a recent study showed that CD152 signalling does not simply silence T cells but endows their capacity to migrate to sites of infection and secondary lymphoid organs [32], by upregulating the expression of CCR7 on T cells [32]. Therefore, the high expression level of CD152 in MG thymus might be due to the enrichment of CCR7 + CD152 + T cells within the inflamed MG thymus as a consequence of chemokine expressed on specialized lymphatic vessels, for example, the CCR7 ligand CCL21 [44]. The increase of VEGF-A, a growth factor mediating vascular permeability and vasculogenesis [30], and ECE, a metalloprotease implicated in proteolytic processing of endothelial precursors [34], might be associated with the abnormal lymphocyte recruitment and the angiogenic processes occurring in MG thymus [44].

Our LDA data showed increased expression of CD19, marker of B cells, in each MG thymus subgroup, thus reflecting the presence of significant B cell infiltration in MG thymus; these results are in line with previous microarray data obtained from Le Panse and colleagues [15].

We also found increased levels of complement component C3 mRNA in MG compared to normal thymuses (Table 2), consistent with previous observation of persistent complement attack on AChR-expressing thymic epithelial and myoid cells in MG hyperplastic thymus; this attack might be responsible for an increased level of autoantigen presentation to dendritic cells sustaining autoimmune reaction [45].

To confirm the MG thymic inflammatory state suggested by LDA data, we performed real-time PCR analysis of six genes, selected for playing key roles in inflammation and host defence responses against infections, in a total of 27 MG (Patient Groups 1 and 2) and 7 control thymuses. These genes were IL-6, IL-10, IFN-*γ*, and HLA-DR*α*, previously analysed by LDA, and IFN-*β* and MxA. IFN-*β* was chosen to search for evidence of action of type I IFNs in MG thymus, as this type of IFNs plays key roles in host immune response against viral infections and has been widely implicated in autoimmune conditions [46]; MxA was investigated as it is an important mediator of type I IFNs in the innate antiviral response [35].

Real-time PCR analysis confirmed upregulation of IL-6, IL-10, IFN-*γ*, and HLA-DR*α* in MG thymuses compared to controls (Figure 1). Interestingly, IFN-*β* and MxA genes were also overexpressed in MG thymuses, supporting the hypothesis of an ongoing antiviral and inflammatory response in MG pathological tissues. Overexpression of IFN-*γ* and IFN-*β* was in agreement with previous data [14] showing that large number of type I and type II IFN-induced genes were significantly upregulated in hyperplastic MG thymuses compared to controls. Previous transcriptional profile analysis of thymus from untreated and steroid-treated MG patients showed that the inflammatory state was reduced upon treatment [47]; in particular, the expression of type I IFN-induced genes, but not of type II IFN-induced genes, was normalized, suggesting that inflammation downmodulation by steroids occurs through type I IFN-pathways [47]. Our thymic transcriptome analysis by LDA and real-time PCR underlines a generalized thymic inflammatory state in MG patients, with increased expression of inflammatory genes being observed even for patients treated with corticosteroid before thymectomy (Table 2, Patient Group 1 and 2). This suggests that inflammatory condition does not completely disappear or is maintained after immunosuppressive treatment. However, the number of steroid-untreated patients we analysed were low (2/10 patients in LDA and 5/27 in real-time PCR analysis); thus, further studies are needed to understand whether immunosuppressive treatment is able to reduce the MG intrathymic proinflammatory condition and establish whether other genes, besides type I IFN-induced genes [47], undergo normalization.

The overall results of our transcriptional profiling confirm that MG thymus is characterized by a chronic inflammatory state. Whether this state is the consequence of viral infection events remains to be clarified. Our previous study showing increased expression of TLR 4—key member of innate immunity—in MG thymuses with thymitis and thymus involution [39], together with the finding of a persistent poliovirus infection in the thymus of some MG patients [16], strongly supports a role of viral infections and innate immune system activation as trigger events for inflammation and intrathymic autosensitization in MG.

### 4.2. EBV Infection Is Commonly Found in MG Thymus

In our previous study, we demonstrated active EBV infection in 17/17 nonneoplastic MG thymuses investigated, irrespective of thymic pathology, whereas no evidence of EBV infection was found in 6 control thymuses from adult healthy subjects [6]. Specifically, in the MG thymuses analyzed, we found (a) a high frequency of EBV-infected B cells by in situ hybridization for EBERs and immunohistochemistry for latent (EBNA2, LMP1, LMP2A) and lytic (BFRF1, BMRF1, gp350/220, p160) EBV proteins; (b) expression of latent (EBNA1, LMP2A) and lytic (BZLF1) genes by nested PCR reactions on cDNA; (c) presence of EBV DNA by real-time PCR specific for LMP1 gene [6]. 

In the present study, we addressed whether EBV infection is a characteristic feature of MG thymus by extending our search for EBV-associated nucleic acids and proteins in additional 19 MG thymuses (Patient Group 3). We decided to apply different molecular approaches from those previously used [6], in order to verify whether we were equally able to detect EBV DNA and RNA in MG thymus (see Section 2). 

Consistent with our previous findings [6], all 19 MG thymuses investigated showed signs of EBV infection (Table 3). EBV DNA was detected in 12/19 MG thymuses (Figure 3 and Table 3); EBV latent or lytic transcripts (often both) were present in all, except one (MG12), MG thymuses, whereas no sign of infection was found in two nonpathological controls (Figure 4 and Table 3). The one MG sample (MG12) negative for EBV transcripts, but harbouring infiltrating B cells by immunohistochemistry, had detectable EBV DNA genome, suggesting that the degree of EBV infection in this thymic specimen could be low (or confined to few cells). 

EBV-encoded RNA called EBER1 is expressed at high levels in EBV-infected cells during latency [17, 18]; most MG patients were positive to EBER1 (Figure 4), consistent with results of our previous study in which the use of in situ hybridization for EBERs allowed us to identify a high proportion of EBERs-positive cells in most of the examined MG thymuses irrespective of the thymic pathology [6]. Here, the application of real-time RT-PCR to detect EBER1, as well as the use of independent real-time PCR assays to detect EBV DNA, LMP1, and EBNA1, strongly confirm evidence for EBV latency in MG thymus. 

To establish latent infection, EBV uses four different latency gene programs (latency III, II, I, and 0), each characterized by expression of a set of viral genes that provide activation, growth, and survival signals to infected B cells [17, 18]. In this study, we detected in MG thymuses EBNA1, which is expressed in all EBV latency programs, and LMP1, which is expressed in latency III (or growth program) and latency II (or default program). In our previous study [6], we searched also for LMP2A (both transcript and protein), which is expressed in latency III and II, and EBNA2 (protein), the first latency protein to be synthesized after infection of naïve B cells and expressed only in latency III (or growth program) [17, 18]. We detected LMP2A, whereas EBNA2 was rarely detected, being identified only in rare cells in few MG thymuses, likely newly infected cells [6]. These previous results, together with those presented here, seem to suggest that EBV mainly uses the latency II to establish a latent infection in MG thymus.

Of the EBV lytic genes, we analyzed the immediate early lytic gene BZLF1, which encodes a transactivator protein regulating expression of early lytic genes [17, 48]. Consistent with our previous results [6], BZLF1 transcript was detected in most (16/19) of the examined pathological thymuses but in none of controls (Table 3 and Figure 4), indicating productive viral infection in MG thymus that may result in new infection events and propagation of EBV infection within MG thymus.

We demonstrated that our real-time PCR analysis could detect EBV transcripts in RNA extracted from a single JY EBV-infected cells (Figures 3 and 4). However, we were unable to detect all the viral transcripts analysed in all the MG thymuses investigated, although all patients were positive for at least one transcript. As suggested by Aloisi and colleagues [49], this may be due to the fact that the quality of RNA extracted from fragments of bioptic tissue sample cannot be exactly compared with that of viable, highly replicating lymphoblastoid cells that contain multiple copies of EBV genome and display high transcriptional activity. Moreover, successful detection of EBV nucleic acids within a highly heterogeneous cell population, which is a feature of a human tissue, may be difficult to achieve [49].

To confirm at the protein level the results of molecular analysis, we performed immunostaining for LMP1 and BZLF1, a latent and a lytic marker (Table 4). As in our previous study [6], we found numerous LMP1-expressing cells in the thymic medulla of MG thymuses with hyperplasia, thymitis, and thymic involution, in areas corresponding to lymphoid infiltrates and (in hyperplasia) to GCs (Figure 6). LMP1 was not detected in the normal thymuses analysed (Figure 6). In the same tissue, immunohistochemistry also revealed the presence of cells positive to BZLF1 in each MG thymic subgroup but in none of the control thymuses (Figure 6). LMP1 and BZLF1 proteins were not detected in 2 of the 8 MG thymuses analysed (LMP1 in MG11 and BZLF1 in MG13), in which the corresponding transcript was also not detected. 

Most of the examined patients (12/19) underwent immunosuppressive therapy before thymectomy (Table 1). Of the remaining 7 patients, 6 (MG1, MG3, MG5, MG8, MG9, and MG13) were only treated with acetylcholinesterase inhibitors and one (MG4) was untreated. We found evidence of EBV infection also in the thymus of these 7 patients, thus suggesting that intrathymic EBV dysregulation is not the consequence of immunosuppressive therapy. However, we cannot exclude that immunosuppressive drugs could amplify an established intrathymic EBV infection.

In conclusion, the findings here presented strengthen the idea that EBV is implicated in the intrathymic pathogenesis of MG. EBV infection might result in the maintenance of the autoimmune response in MG thymus by contributing to chronic B cell activation and promoting survival and expansion of autoreactive B cell clones. Whether active intrathymic EBV infection is a primary event in MG or the consequence of an underlying intrathymic process that results in both attraction of circulating EBV-infected cells and EBV reactivation in MG thymus needs to be clarified. The high prevalence of EBV infection in the population and low incidence of MG suggests that other factors (genetic or environmental, or both) must intervene together with EBV to cause MG. It is possible that a preexisting inflammatory state might be necessary for colonization of thymus by EBV-infected B cells and subsequent reactivation of these cells, and in turn a chronic EBV infection itself might play a role in sustaining chronic intrathymic inflammation in MG creating a vicious circle.

## 5. Conclusions

Inflammation is an important contributor factor in the development and progression of autoimmune diseases. The results of transcriptional profiling here presented, by confirming previous data showing inflammation and active immune response in MG thymus, strongly support the idea that the creation of a local proinflammatory state is a pathogenic feature of thymus in MG patients. 

**Figure 7:**
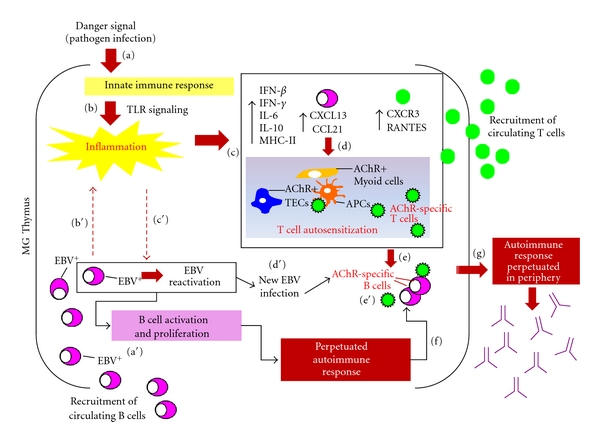
Proposed model of virus-induced autoimmunity in MG. A “danger signal” (e.g., pathogen infection) stimulates Toll-like receptor-(TLR-) mediated innate immune responses (a), whose dysregulated or persistent activation leads to the chronic inflammation characteristic of MG thymus (b). The chronically established thymic inflammatory state (c), characterized by overexpression of proinflammatory cytokines (e.g., IL-6, IL-10), type I and II IFNs, and T- and B-lymphocyte-attracting chemokines (e.g., CXCR3, RANTES, CXCL13, CCL21), is essential, in the context of a genetically predisposing background, for the establishment of mechanisms (d) contributing to T-cell autosensitization, including presentation or cross-presentation of “self-epitopes” by TECs or myoid cells expressing the autoantigen; upregulation of MHC genes; activation of antigen-presenting cells (APCs); as well as the constant priming of autoreactive T cells, which in turn promote autoimmune response by autoreactive B cells (e). B cell attractants CXCL13 and CCL21 recruit circulating B cells to thymus, including those harboring EBV (a′). EBV infection itself contributes to thymic inflammation (b′). EBV reactivation, influenced by the inflammatory state (c′), results in EBV propagation to uninfected B cells (d′) including AChR-specific B cells (e′). The chronically established inflammation and EBV infection promote the maintenance within the thymus of the autoimmune response (f), which may be thus perpetuated in periphery (g).

We postulate that a chronically established thymic inflammation may be essential, in the context of a genetically predisposing background, for the establishment of mechanisms contributing to MG autoimmunity including presentation of “self-epitopes”; upregulation of MHC genes, of type I IFNs, of proinflammatory cytokines, of adhesion, and of costimulatory molecules on antigen-presenting cells; as well as the constant priming of autoreactive T cells [13] (Figure 7). Evidence of persistent viral presence in MG thymuses, derived from our recent studies [6, 16], suggests that an initial pathogen infection might be responsible for the observed inflammatory signature and the subsequent autoantigen sensitization in MG thymus. In particular, our recent finding [6], here confirmed and reinforced, of an active EBV infection in the intrathymic B cell component in MG patients suggests that EBV infection, together with inflammation, may be a key step in the intrathymic pathogenesis of MG. Inflammation triggered by an endogenous or exogenous (e.g., microbial infection) danger signal may drive the colonization of thymus by EBV-harbouring B cells and the subsequent EBV reactivation (Figure 7). Persistent EBV infection itself may contribute to maintain a chronically inflamed thymic microenvironment. In the inflamed thymus, EBV may promote disruption of B-cell tolerance checkpoints and result in expansion of autoreactive B cell clones (Figure 7). EBV infection thus could explain how the autoimmune response can be perpetuated in MG thymus, since EBV is potentially able to immortalize B cells that are producing AChR antibodies.

By adding new evidence for inflammation and EBV infections as common feature of MG thymus, our findings may have relevant therapeutic implications: they reinforce the rationale for current therapeutic approaches, particularly anti-inflammatory drug use and thymectomy to remove the site of infection, and also suggest future rationale preventive and therapeutic measures for MG, such as EBV vaccination [50] or regulation of the existing EBV infection by the use of antiviral agents.

## Figures and Tables

**Table 1:** Clinical features of MG patients included in the study.

Patient Group 1	Hyperplasia (*n* = 3)	Thymitis (*n* = 3)	Involuted (*n* = 4)
Sex (F/M)	3/0	2/1	2/2
Age at disease onset (years) mean ± SD	23.3 ± 9.3	30.9 ± 3.7	29.1 ± 16.3
Age at surgery (years) mean ± SD	26.3 ± 11.0	32.0 ± 3.6	31.5 ± 15.2
Ab AChR-positive	2	3	4
Seronegative	1	0	0
Immunosuppressive therapy	2	2	4

Patient Group 2	Hyperplasia (*n* = 6)	Thymitis (*n* = 6)	Involuted (*n* = 5)

Sex (F/M)	5/1	5/1	4/1
Age at disease onset (years)^a^ mean ± SD	23.6 ± 7.2	23.4 ± 12.6	30.0 ± 10.2
Age at surgery (years) mean ± SD	25.5 ± 6.0	25.2 ± 11.9	33.0 ± 11.2
Ab AChR-positive	6	4^b^	4
Seronegative	0	1	1
Immunosuppressive therapy	5^c^	4	5

Patient Group 3	Hyperplasia (*n* = 9)	Thymitis (*n* = 5)	Involuted (*n* = 5)

Sex (F/M)	8/1	4/1	3/2
Age at disease onset (years)^d^ mean ± SD	26.4 ± 9.2	31.0 ± 3.8	30.7 ± 14.4
Age at surgery (years) mean ± SD	30.0 ± 10.9	33.4 ± 4.0	32.20 ± 11.8
Ab AChR-positive	6	4	5
Seronegative	3	1	0
Immunosuppressive therapy	3	4	5

^a^Age at disease onset was not available for two patients, one with hyperplasia and one with thymitis. ^b^Information on autoantibody presence in serum was not available in one patient. ^c^Data on the therapy before thymectomy were missing in one patient. ^d^Age at disease onset was not available for two patients, one with hyperplasia and one with involuted thymus.

**Table 2:** Upregulated genes in follicular hyperplasia, thymitis, and involuted thymus versus normal thymus identified by TaqMan low-density arrays (LDAs).

Gene symbol	Biological function	Fold changes^a^		
		Hyperplasia	Thymitis	Involuted
CSF1	Cytokine	3.61 (1.23)	7.16 (2.78)*	2.22 (1.15)
IL-1*β*	Cytokine	11.29 (13.44)	51.72 (30.57)*	10.92 (10.92)
**IL-6**	Cytokine	29.56 (14.44)	1621.00 (513.70)**	72.60 (20.25)**
**IL-10**	Cytokine	5.93 (0.41)*	7.64 (1.12)*	7.14 (0.61)**
IL-12p35	Cytokine	4.09 (3.47)	15.42 (6.55)*	3.42 (2.11)
TNF-*α*	Cytokine	2.97 (1.61)	8.92 (2.95)*	3.27 (1.10)
**IFN-*γ***	Cytokine	2.21 (0.78)	15.24 (4.89)*	1.78 (2.30)
RANTES	Cytokine	4.90 (1.07)*	6.79 (1.26)*	3.31 (0.95)
IL-7	Growth factor	5.25 (2.27)	21.18 (8.36)*	2.78 (0.92)
VEGF-A	Growth factor	5.01 (2.57)	20.07 (9.74)*	4.62 (3.02)
IL-8	Chemokine	3.90 (3.25)	16.22 (5.61)*	3.57 (2.00)
CXCR3	Chemokine receptor	4.56 (0.50)*	6.15 (1.19)*	2.57 (0.70)
MCP-1	Chemokine	4.41 (1.36)	31.08 (5.68)*	20.53 (1.60)**
MIP-1*α*	Chemokine	17.93 (11.38)	35.01 (11.34)*	16.11 (7.89)
CD19	CD antigen	4.37 (0.17)*	6.91 (1.41)*	3.51 (1.25)
CD86	CD antigen	1.82 (0.09)	3.82 (1.52)*	1.05 (0.44)
CD152	CD antigen	3.38 (0.49)	7.87 (3.37)*	2.97 (1.77)
**HLA-DR*α***	MHC Class II	2.12 (0.39)	4.15 (1.05)*	1.58 (0.78)
C3	Complement component	10.66 (4.75)	29.12 (6.87)*	12.83 (10.88)
SMAD7	Cell signaling	2.53 (0.87)	4.78 (0.62)**	1.61 (1.06)
ECE1	Metalloprotease	2.57 (0.97)	6.34 (2.51)*	2.42 (1.51)

^a^For each gene, mean fold change (±SD) for the different MG thymus subgroups compared to normal thymuses is given. Fold change was calculated from the formula 2^−∆∆Ct^; *P < 0.05, **P < 0.01 (Bonferroni test). The genes in bold were further analysed by real-time RT-PCR.

**Table 3:** Detection of EBV DNA and RNA transcripts in MG thymus by real-time PCR.

Patient	Thymic pathology	Anti-AChR antibodies^a^	EBV DNA^b^	Latent markers^b^			Lytic marker^b^
				EBER1	EBNA1	LMP1	BZLF1
MG1	Hyperplasia	Positive	+	+	+	−	+
MG2	Hyperplasia	Positive	+	+	+	+	+
MG3	Hyperplasia	Positive	−	+	+	−	−
MG4	Hyperplasia	Positive	−	+	+	−	+
MG5	Hyperplasia	Positive	+	+	+	+	+
MG6	Hyperplasia	Positive	+	+	+	+	+
MG7	Hyperplasia	SN	+	−	−	+	+
MG8	Hyperplasia	SN	−	−	+	−	+
MG9	Hyperplasia	SN	+	+	+	−	+

MG10	Thymitis	Positive	−	−	−	+	+
MG11	Thymitis	Positive	−	+	+	−	+
MG12	Thymitis	Positive	+	−	−	−	−
MG13	Thymitis	Positive	+	+	+	−	−
MG14	Thymitis	SN	−	−	+	+	+

MG15	Involuted	Positive	+	+	−	−	+
MG16	Involuted	Positive	+	+	+	+	+
MG17	Involuted	Positive	+	+	+	+	+
MG18	Involuted	Positive	+	+	+	+	+
MG19	Involuted	Positive	−	+	+	−	+

Ctr1^c^	Normal	SN	−	−	−	−	−
Ctr2^c^	Normal	SN	−	−	−	−	−

^a^SN: seronegative (AChR- and MuSK-negative) patients. ^b^EBV genome and transcripts were analysed by real-time PCR techniques as described in Section 2. Results are expressed as follows: + detected (Ct value <38); − not detected (Ct value >38). ^c^Ctr: nonpathological control thymus.

**Table 4:** Detection of EBV latent LMP1 and lytic BZLF1 proteins in MG thymus by immunohistochemistry.

Patient	Thymic	Anti-AChR	EBV proteins^b^	
	pathology	antibodies^a^	LMP1	BZLF1
MG1	Hyperplasia	Positive	+	+
MG5	Hyperplasia	Positive	+	+
MG6	Hyperplasia	Positive	+	+
MG9	Hyperplasia	SN	+	+

MG11	Thymitis	Positive	−	+
MG13	Thymitis	Positive	+	−

MG16	Involuted	Positive	+	+
MG17	Involuted	Positive	+	+

Ctr1^c^	Normal	SN	−	−
Ctr2^c^	Normal	SN	−	−

^a^SN: seronegative (AChR- and MuSK-negative) patients. ^b^Results of immunostaining for LMP1 and BZLF1 are expressed as follows: + presence of positive cells; − absence of positive cells. ^c^Ctr: nonpathological control thymus.
